# Repurposing the anthelmintic drug niclosamide to combat *Helicobacter pylori*

**DOI:** 10.1038/s41598-018-22037-x

**Published:** 2018-02-27

**Authors:** Nagendran Tharmalingam, Jenna Port, Dawilmer Castillo, Eleftherios Mylonakis

**Affiliations:** Infectious Diseases Division, Warren Alpert Medical School of Brown University, Rhode Island Hospital, Providence, RI 02903 USA

## Abstract

There is an urgent need to discover novel antimicrobial therapies. Drug repurposing can reduce the time and cost risk associated with drug development. We report the inhibitory effects of anthelmintic drugs (niclosamide, oxyclozanide, closantel, rafoxanide) against *Helicobacter pylori* strain 60190 and pursued further characterization of niclosamide against *H. pylori*. The MIC of niclosamide against *H. pylori* was 0.25 μg/mL. Niclosamide was stable in acidic pH and demonstrated partial synergy with metronidazole and proton pump inhibitors, such as omeprazole and pantoprazole. Niclosamide administration at 1 × MIC concentration, eliminated 3-log_10_ CFU of *H. pylori* adhesion/invasion to AGS cells. Interestingly, no resistance developed even after exposure of *H. pylori* bacteria to niclosamide for 30 days. The cytotoxic assay demonstrated that niclosamide is not hemolytic and has an IC_50_ of 4 μg/mL in hepatic and gastric cell lines. Niclosamide administration decreased transmembrane pH as determined by DiSC_3_(5) assay indicating that the mechanism of action of the anti-*H. pylori* activity of niclosamide was the disruption of *H. pylori* proton motive force. Niclosamide was effective in the *Galleria mellonella*-*H. pylori* infection model (*p* = 0.0001) and it can be develop further to combat *H. pylori* infection. However, results need to be confirmed with other *H. pylori* and clinical strains.

## Introduction

*Helicobacter pylori* is a Gram-negative, helically shaped, stomach pathogen associated with human gastric mucosa. This bacillus can cause chronic gastritis, peptic ulcer, gastric mucosa-associated lymphoid tissue (MALT) lymphoma and gastric carcinoma^1^. About 2.9% of *H. pylori* infected individuals develop gastric cancer^2^ and eradication of *H. pylori* infection decreased the risk of gastric cancer^2,3^. Moreover, even though the association between the *H. pylori* infection and colorectal cancer is inconclusive^4^, meta-analysis by Wu *et al*., demonstrated that the *H. pylori* infection was associated with increased occurrence of colorectal cancer^5^. *H. pylori* infection is often initiated in childhood^6^. This results in an ongoing *H. pylori* infection in about half of the global population^7^. The World Health Organization (WHO) declared *H. pylori* a class I gastric carcinogen^8^ with worldwide prevalence, and increased impact in low socio-economic countries^9^. Successful antimicrobial treatment for *H. pylori* infection is extremely challenging due to its survival in a hostile acidic environment and association within the gastric mucosa^10^. As such, the antimicrobial agents must penetrate thick mucus, remain active in low pH and a successful therapy requires sequential administration of two antibiotics, such as amoxicillin and clarithromycin, along with a proton pump inhibitor (PPI)^10^. However, traditional antibiotics are failing to clear *H. pylori* infection at ever increasing rates, primarily due to the emergence of bacterial drug resistance^11^. For example, *H. pylori* resistance to clarithromycin among male U.S. veterans increased from 16% to 24% during 2009 to 2013 and resistance to metronidazole is high^11^.

Similar to the difficulties with antimicrobial drug discovery in general, development of novel anti-*H. pylori* agents has been challenging due to regulatory guidelines, depressed financial returns, and difficulties determining the mechanism of action of novel compounds^12^. Repurposing is a powerful approach for identifying potential antimicrobial characteristics of existing clinical molecules that have been prescribed for other therapies in healthcare. Research from others, as well as our group, previously reported the antibacterial activities of anthelmintics on MRSA^13,14^, *Clostridium difficile*^15^ and *Pseudomonas aeruginosa* quorum sensing^16^. Anthelmintics are used to treat protozoan-helminth infections in humans and they are among the most used and lowest-priced medicines world-wide^17^. Niclosamide is primarily used to treat cestodes (tapeworms) such as *Taenia saginata*, *Taenia solium, Hymenolepsis nana*^18^ and it inhibits production of energy derived from anaerobic metabolism^19^. Niclosamide, is a known anthelmintic that is listed in the WHO essential medicines^20^, and we reported that the drug was active against *H. pylori* infection. This manuscript further investigated whether they possess activity against *H. pylori* strain 60190, both *in vitro* and *in vivo*.

## Results and Discussion

### Antibacterial susceptibility

We conducted a pilot screening for anti- *H. pylori* activity by a broth microdilution assay using the hit molecules that was identified from our previous HTS^14,21^, which can be found in the Supplementary Table. S2. Interestingly, we found that the anthelmintics (niclosamide, oxyclozanide, rafoxanide, and closantel) inhibited the growth of *H. pylori* strain 60190. More specifically, the MICs of niclosamide, oxyclozanide, rafoxanide, and closantel were 0.25, 2.0, 4.0 and 16 μg/mL, respectively (Table 1**)** and the MBC of niclosamide, oxyclozanide, rafoxanide, and closantel against *H. pylori* were 0.5, 4.0, 8.0, 32 μg/mL, respectively (Table 1**)**.

**Table 1:** Antimicrobial properties of anthelmintics against *H. pylori*.

Antimicrobial agent	MIC μg/mL	MBC μg/mL
Niclosamide	**0.25**	**0.5**
Oxyclonazide	**2.0**	**4.0**
Rafoxanide	**4.0**	**8.0**
Closantel	**16**	**16.0**
Amoxicillin	**0.01**	**0.5**
Clarithromycin	**0.0025**	**0.25**

**Figure 1:**
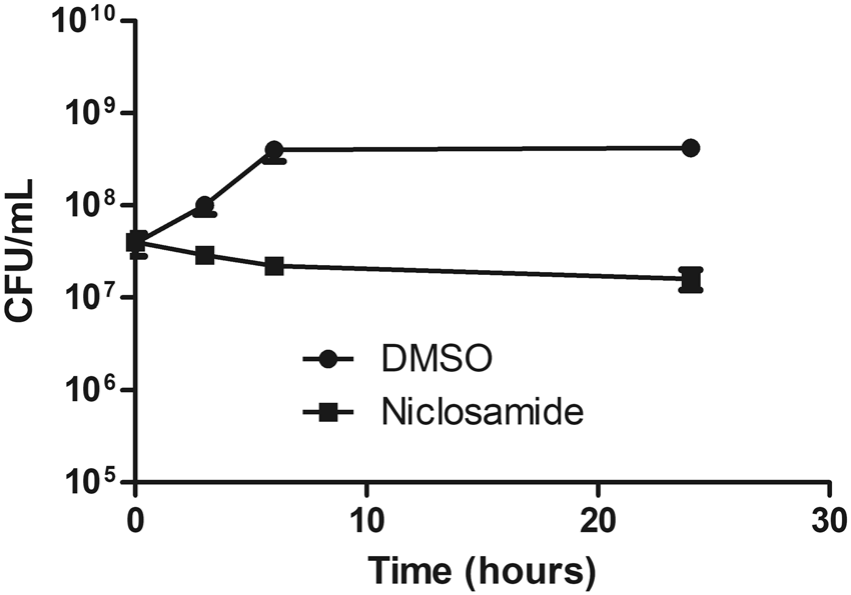
Time to kill assay. Killing kinetics of niclosamide on *H. pylori* at 4× MIC (1 μg/mL) was determined by a time to kill assay and was found to be bacteriostatic.

The MIC of niclosamide was the lowest of the anthelmintics tested (0.25 μg/mL, Table 1), therefore we focused on this agent and time to kill assays were used to confirm the killing properties of niclosamide against *H. pylori*. Bacterial cells (10^9^/mL) exposed to the compounds at 4× MIC showed potent inhibition of the bacterial cell division relative to DMSO controls (Fig. 1). The finding that niclosamide is bacteriostatic against *H. pylori* is consistent with previous data reported by our research group claiming that niclosamide is bacteriostatic against MRSA^13^. Of note, clarithromycin, a commonly prescribed antibiotic for *H. pylori* treatment, is also bacteriostatic^22^.

### Niclosamide partially synergized with clinical molecules

Use of multiple antimicrobial agents can decrease bacterial resistance and even re-establish the clinical efficacy of certain antibiotics^23^. Checkerboard assays were carried out to determine whether niclosamide can act synergistically against *H. pylori* when paired with clinical antibiotics and PPIs. Paired combinations of compounds and their observed fractional inhibitory concentration indices (FICI) are listed in Table 2. Synergistic activity, where the combined antibacterial effect of the 2 antimicrobial agents is more than the sum of their effects alone, is defined by FICI ≤ 0.5, antagonism by FICI > 4.0 and ‘no interaction’ by FICI > 0.5–4.0^24^. In our assays, antagonism was not observed for any of the combinations (amoxicillin, clarithromycin, metronidazole, omeprazole, and pantoprazole) with niclosamide. Niclosamide was partially synergistic with metronidazole, and the PPIs omeprazole and pantoprazole (Table 2). Notably, PPIs are known for their role in decreasing acid secretion, but they do exhibit mild anti-*H. pylori* activity^25,26^. The main purpose of PPIs in triple therapy (two antibiotics with one PPI) is to potentiate the antibacterial activity of antibiotics in a hostile gastric acid environment^27^. Taken together, our results indicate that niclosamide is highly active in acidic pH and can also partially synergize with omeprazole and pantoprazole (Table 2).

**Table 2:** FICI value of niclosamide with other test compounds.

Test compounds	FICI value Niclosamide
Amoxicillin	**2.0**
Clarithromycin	**1.0**
Omeprazole	**0.625**
Metronidazole	**0.75**
Pantoprazole	**0.75**

Synergy FICI ≤ 0.5, partial synergy 0.5 < FICI ≤ 1.0, no interaction 1.0 > FICI ≤ 4.0, antagonism FICI > 4.0.

### Inhibitory role on adhesion/invasion

**Figure 2:**
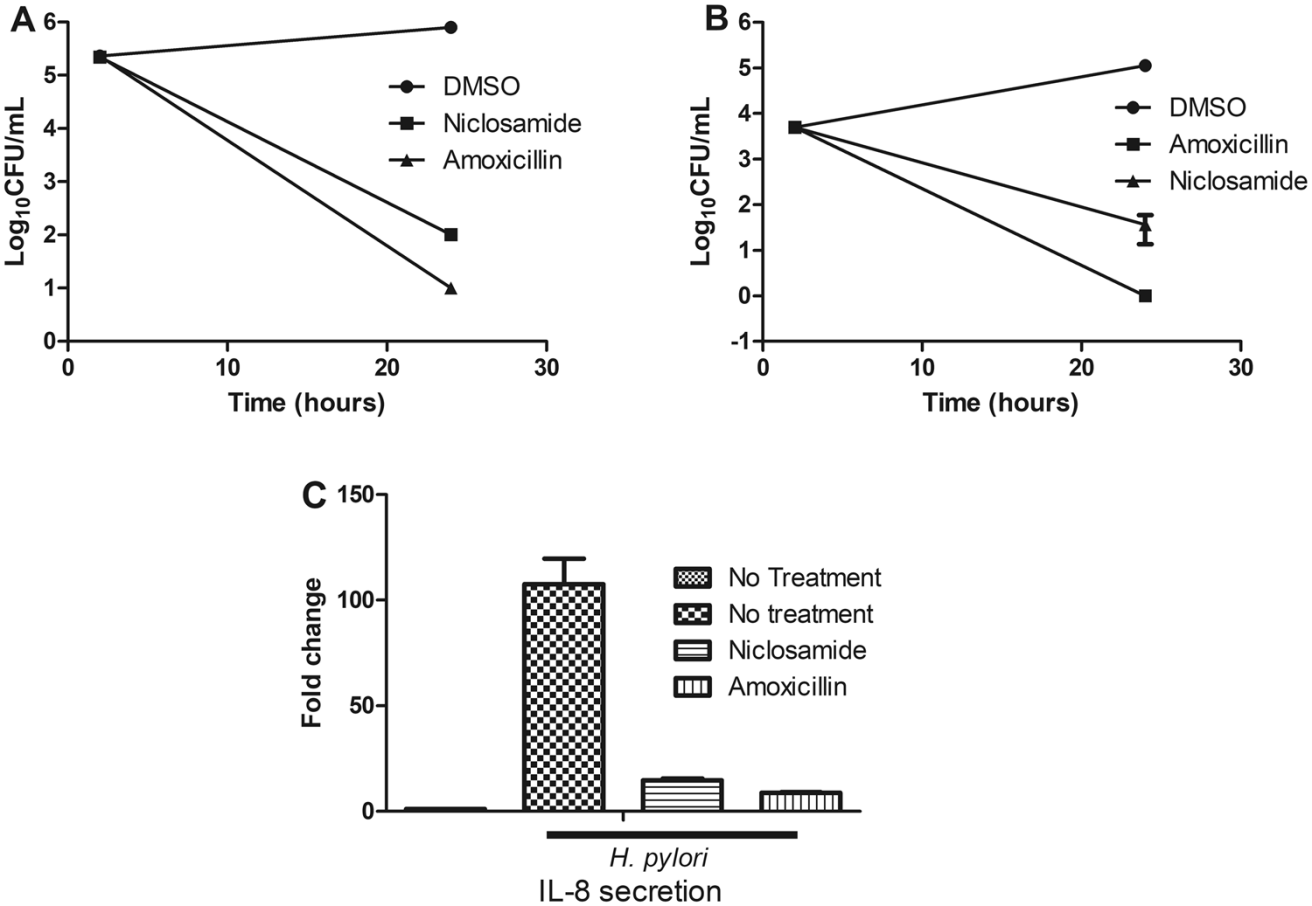
*In vitro* infection assay. (**A**) Adhesion assay. Niclosamide removed adhered *H. pylori* on AGS at a concentration of 2× MIC (0.5 μg/mL). (**B**) Invasion assay Niclosamide prevented *H. pylori* invasion into AGS at a concentration of 2× MIC (0.5 μg/mL). Data represent the mean ± SEM (n = 3). **(C**) Real-Time PCR. Niclosamide (at 1.0 μg/mL that is 4× MIC) inhibited *H. pylori-*induced IL-8 secretion in MKN-28 cells.

Adhesion is the initial step in the *H. pylori* infection process^28^. In order to determine the role of niclosamide on adhesion, we allowed *H. pylori* to adhere to AGS cells for 2 h then administrated niclosamide for 24 h (2× MIC, 0.5 μg/mL). After incubation, we observed that the administrated concentration eliminated the adhered bacteria and reduced CFU/mL counts by 3-log_10_ (Fig. 2A). *H. pylori* is also reported as a facultative intracellular bacterium^29^ and it can survive in the plasma membrane of infected epithelial cells^30^. To determine the role of niclosamide on *H. pylori* invasion, a gentamicin protection assay was performed and it revealed that niclosamide (2× MIC, 0.5 μg/mL) reduced 3-log_10_ CFU in AGS cell lines (Fig. 2B). We found a contradiction between the killing kinetics and adhesion/invasion assays indicating that niclosamide appeared bacteriostatic during the killing kinetics and bacteriocidal during adhesion/invasion assays. We hypothesized that the variance in the efficacy of niclosamide could be due to a difference in bacterial cell concentration. The density of bacterial inoculum in the adhesion/invasion assays was lesser than the time-to-kill assay. A previously reported molecule, omeprazole, acted in a similar manner^31^ and it appeared bacteriostatic in high density and bactericidal in a low density of bacteria. In comparison, amoxicillin treatment (0.1 μg/mL) eliminated bacterial adhesion and invasion completely.

### Niclosamide treatment inhibits IL-8 secretion

*H. pylori* infection induces IL-8 secretion^32^-which, in turn, initiates neutrophil chemotaxis and activation, ultimately causing mucosal damage due to the production of reactive oxygen radicals^33^ and IL-8 secretion is an important factor in the immunopathogenesis of peptic ulcers and its relevant role in gastric carcinogenesis^34^. We carried out Real-Time (RT-PCR) to determine whether niclosamide has an inhibitory role in IL-8 secretion. As shown in Fig. 2C, niclosamide treatment (4× MIC, 1.0 μg/mL) during 24 h incubation period inhibited IL-8 secretion in *H. pylori* (MOI = 50) infected MKN-28 cells.

### Stability of niclosamide in acidic pH

**Figure 3:**
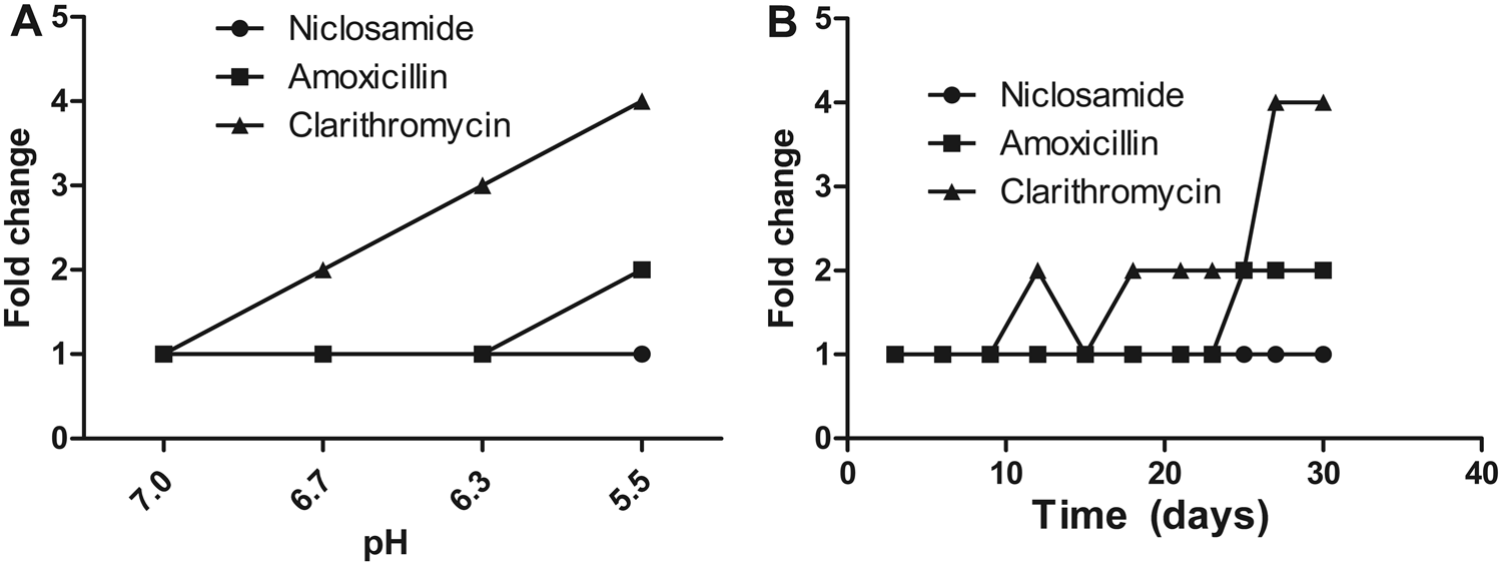
Acid stability of niclosamide and *H. pylori* resistance to antibiotics. (**A**) Niclosamide inhibit *H. pylori* growth at acidic pH. Growth at various pH indicated that MIC of standard antibiotics has increased, but not with niclosamide. (**B**) Mutation frequency of *H. pylori* to niclosamide. *H. pylori* were cultured with clinical antibiotics or niclosamide for 30 days and the emergence of resistance against clinical antibiotics in *H. pylori* was observed, but not with niclosamide. Fold change defined as change in MIC.

In *H. pylori* therapy, antibiotics need to be active in acidic pH^35,36^. However, certain antibiotics lose their potency in low pH^37–39^. Broth microdilution assays in various acidic pHs were carried out to determine the stability of niclosamide. We found that antibacterial potential of niclosamide did not change in acidic pH (tested between 5.0 to 6.5) (Fig. 3A) and the MIC of niclosamide in low pH remained the same as in neutral pH (MIC 0.25 μg/mL).

### Multi-step mutation frequency

*H. pylori* resistance to standard antibiotic treatment is increasing and the resistance rate to clarithromycin is highest in North America and amoxicillin resistance is highest in Africa^40,41^. To determine the potential for resistance, we passaged *H. pylori* treated with either niclosamide or a standard clinical antibiotic (amoxicillin and clarithromycin) for 30 days. The MIC increased 2-fold for clarithromycin and amoxicillin, while that of niclosamide remained constant through at least 10 passages (Fig. 3B). Similarly, the MIC to amoxicillin and clarithromycin increased when *H. pylori* bacteria were exposed to these agents for after 15 days and 24 days, respectively. More specifically, during continuous passage with amoxicillin and clarithromycin, the MIC increased by 2-fold and 4-fold, respectively (Fig. 3B), while MIC to niclosamide remained unchanged, indicating a smaller chance of emerging resistance to niclosamide during *H. pylori* treatment.

### Niclosamide inhibits *H. pylori* motility at sub-MIC level

**Figure 4:**
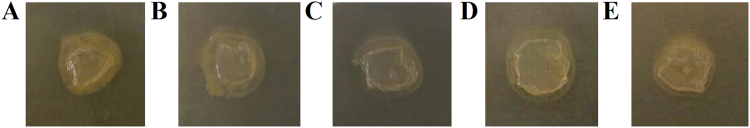
Niclosamide decreased *H. pylori* motility. (**A**–**E**) *H. pylori* was cultured on soft top agar in the presence or absence of niclosamide. Niclosamide treatment impedes *H. pylori* swarming movement in a dose-dependent manner. (**A**) DMSO; (**B**–**E**) niclosamide. (**B**) 75 ng/mL; (**C)** 100 ng/mL; (**D**) 150 ng/mL; (**E**) 200 ng/mL.

Initial colonization of the stomach mucosa by *H. pylori* is associated with the motility of bacteria^42^. In order to evaluate the effects, if any, of niclosamide on *H. pylori* motility, we performed a motility assay and found that motility decreased under niclosamide treatment at a dose-dependent manner (from 0.10 to 0.20 μg/mL). The swarming movement of *H. pylori* bacteria decreased gradually depending on the concentration of niclosamide. At sub-MIC (0.2 μg/mL) concentrations, the motility was abated (Fig. 4A–E).

### Scanning electron microscopic observations

**Figure 5:**
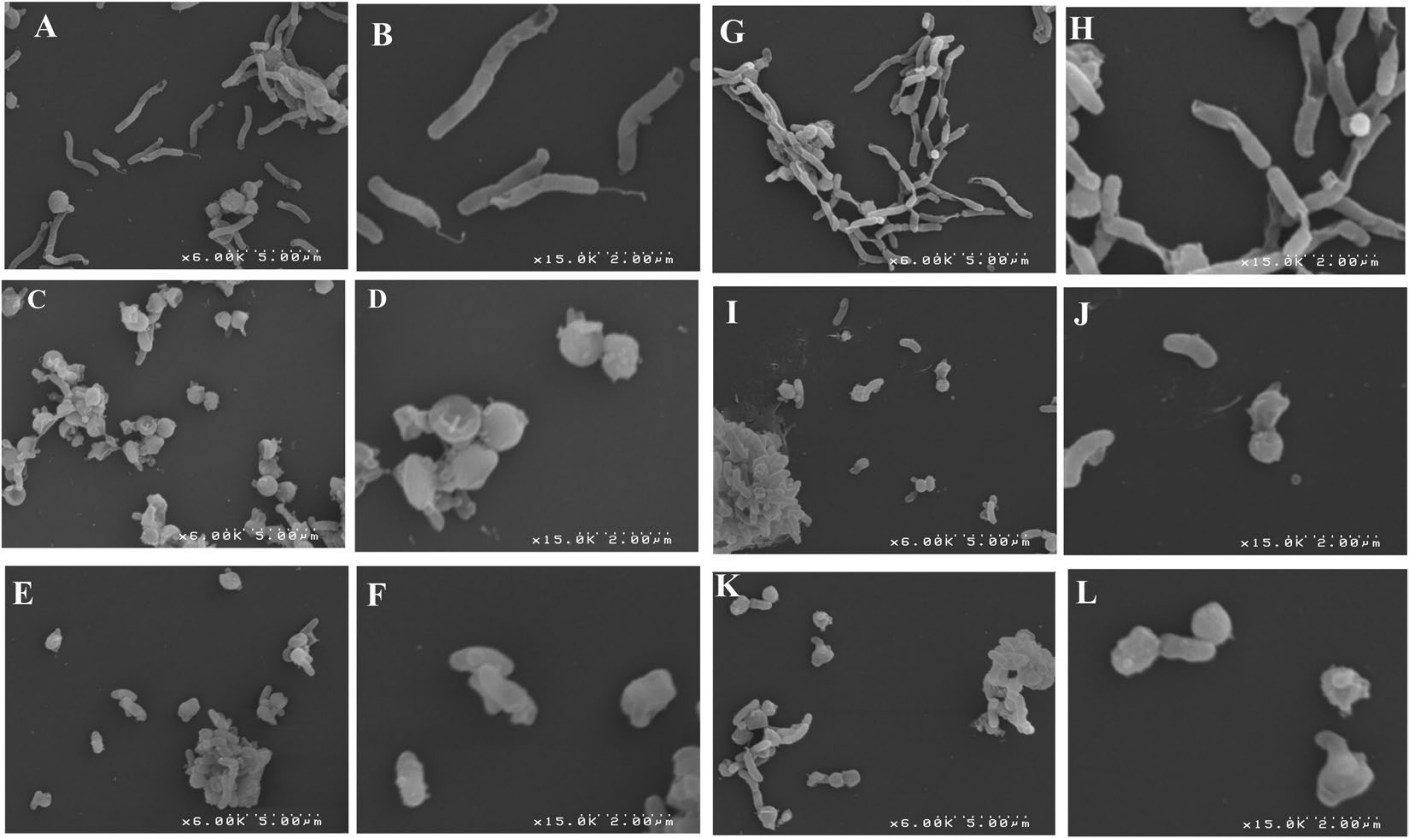
Electron microscopic observations. (**A–L**) *H. pylori* was cultured with or without test drugs for 3 h in a microaerophilic chamber with agitation and morphological changes were observed under the SEM. Niclosamide treatment (1× , 4× , 8× MIC) caused morphological changes such as a shortened length of curved bacilli (**C–H**) compared to no treatment (**A,B**). Amoxicillin (0.1 μg/mL) and CCCP (10 μM) served as control drugs.

To study further the decreased motility, we evaluated the morphology of *H. pylori* bacteria under a scanning electron microscope (SEM) in the presence or absence of niclosamide. The untreated *H. pylori* cells were helically shaped, had intact membrane surface, and possessed well-formed bacterial flagella. However, niclosamide-treated *H. pylori* cells became short bacilli and the morphology changed in a dose-dependent manner (Fig. 5C–H). The helical shape of the bacterium was shortened at 1× MIC (Fig. 5C,D) and appeared to become Cocco-bacillary (Fig. 5E–H) at 4× and 8× MIC. The amoxicillin (0.1 μg/mL) and CCCP (10 μM) groups also exhibited morphological changes and decreased cell size. Interestingly, treatment with niclosamide caused flagellar deformation, similar to that reported by Zhang *et al*.^43^ after exposure to the antimicrobial peptide cathelicidin. This effect in the bacterial flagella may explain the effects of niclosamide in *H. pylori* motility.

### Light microscopy imaging for vacuolation

**Figure 6:**
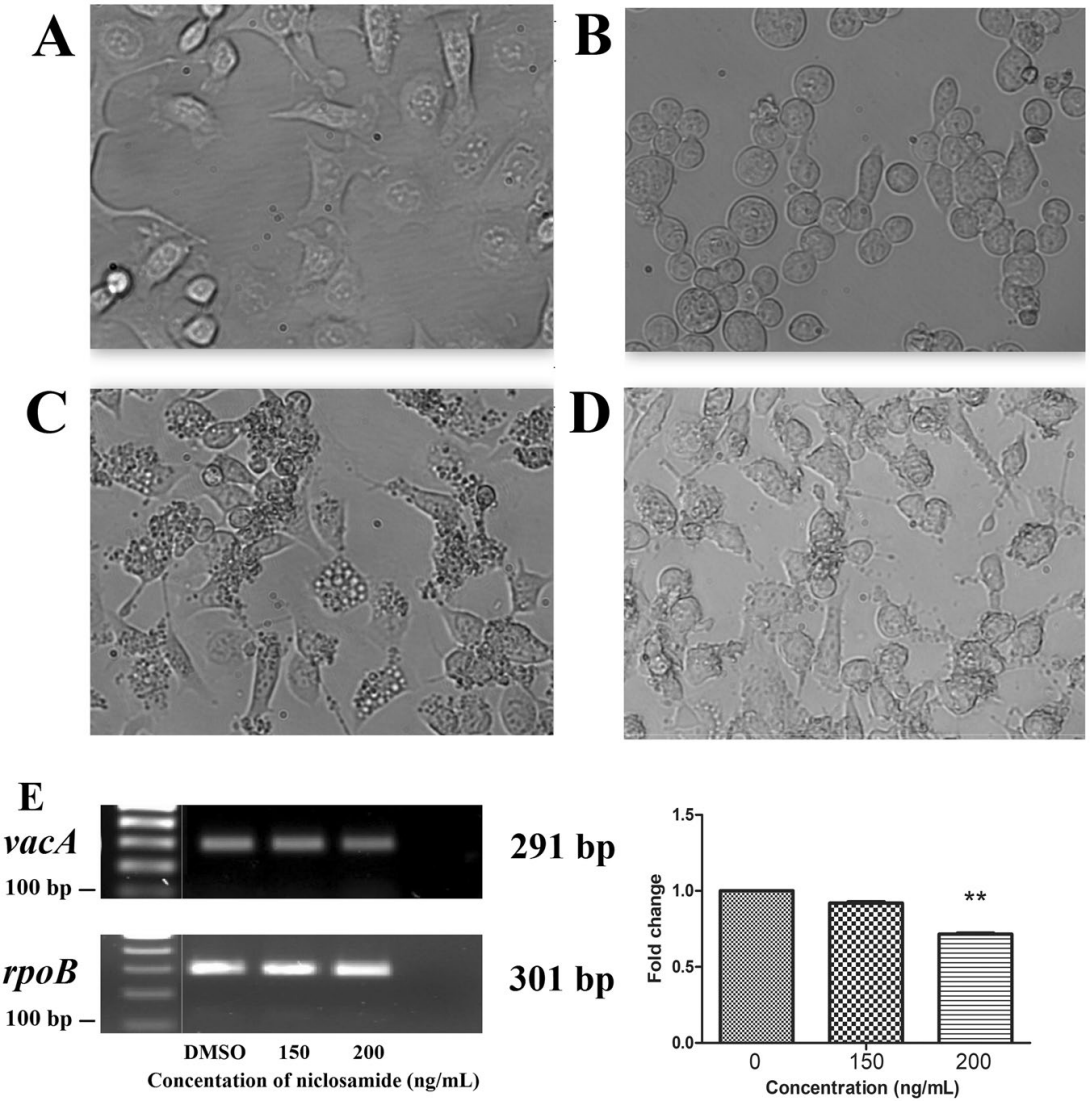
Niclosamide inhibits vacuolation. (**A–D**) AGS cells were infected with *H. pylori* (MOI-100) in the presence or absence of niclosamide and vacuolation were visualized by light microscopy imaging. Results revealed that sub-MIC (0.2 μg/mL) of niclosamide prevented vacuolation. (**A**) AGS alone; (**B**) AGS treated with niclosamide; (**C**) AGS cells infection with *H. pylori*; (**D**) AGS cells cocultured with *H. pylori* and niclosamide. (**E**) Niclosamide down-regulated *vacA* gene (291 bp) expression at sub-MIC (0.2 μg/mL), which is compared to expression of the housekeeping gene *rpoB* (301 bp). The grouping of gels cropped from different parts of the same gel demonstrated by the yellow line drawn on spliced position. Full-length gels are presented in Supplementary Figure S3. ***p < 0.001, students t-test comparing DMSO control.

*H. pylori* bacteria secrete VacA toxin via a type V secretion system^44^. This toxin binds to host gastric epithelial cells and its internalization leads to vacuolation, characterized by the accumulation of large vacuoles in gastric epithelial cells during the infection process^44,45^. To demonstrate whether sub-MIC (0.2 μg/mL) concentrations of niclosamide influences toxin expression in *H. pylori*, RT-PCR was performed. Results indicated that at the sub-MIC level, niclosamide downregulates VacA expression (Fig. 6E). This was further confirmed by the impairment of vacuolation when niclosamide was administered to AGS cocultured with *H. pylori* (Fig. 6A–D).

### Niclosamide role on *H. pylori* proton motive force (PMF)

**Figure 7:**
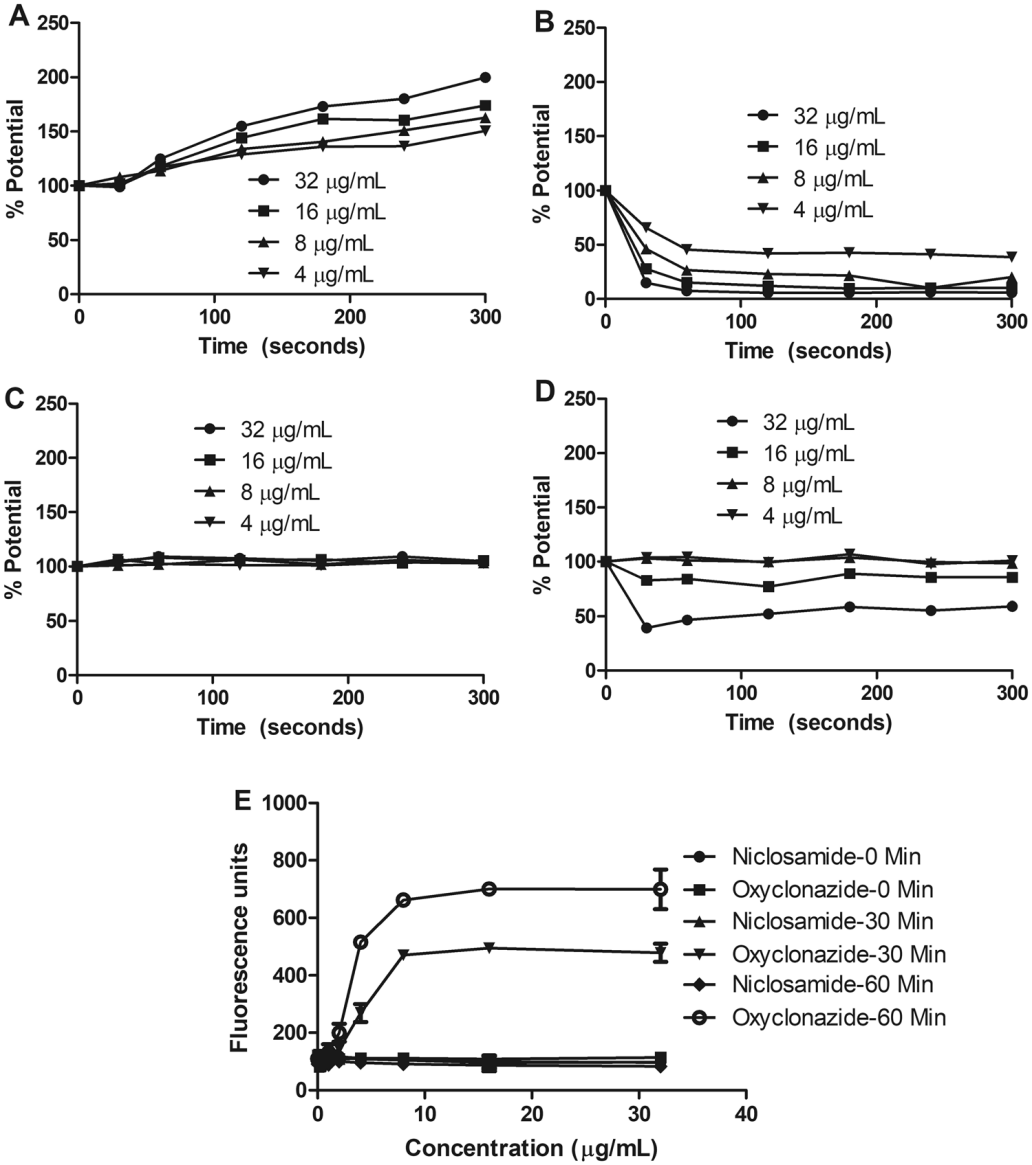
Membrane activity of niclosamide. (**A–D**) Membrane potential. Fluorescence probe Disc3(5) loaded *H. pylori* was treated with (**A**) Valinomycin; (**B**) Niclosamide; (**C**) Amoxicillin; (**D**) CCCP and the fluorescence was recorded before and after treatment. Niclosamide caused decreased transmembrane pH, whereas positive control agent valinomycin perturbed membrane potential. Data depicts three independent experiments. (**E**) Membrane permeability. *H. pylori* was loaded with Sytox green fluorescence probe and 0.5 h later, treated with niclosamide and the cellular fluorescence was measured. Niclosamide did not cause membrane damage of *H. pylori* but the oxyclozanide (positive control) cause membrane damage. Data represent the mean ± SEM (n = 3).

Many antimicrobial agents are known to act on bacterial membranes^46^ and exposure to niclosamide can disrupt the membrane potential^47^. We hypothesized that the mechanism of action of niclosamide is likely to disrupt *H. pylori* PMF. The fluorescent probe DiSC_3_(5) was loaded with *H. pylori* bacteria, which accumulates in the cytoplasmic membrane. The dissipation of PMF can be determined by either increase in fluorescence (disruption of membrane potential) or decrease in fluorescence (dissipation of transmembrane pH)^48^. We found that niclosamide treatment decreased the transmembrane pH of *H. pylori* (Fig. 7B), as determined by decreased fluorescence. In this series of experiments, we used the known protonophore, CCCP, that disrupts PMF by decreasing transmembrane pH^49^ as a positive control (Fig. 7D). Valinomycin (Fig. 7A), that can disrupt membrane potential, and amoxicillin (Fig. 7C), that has effects on the membrane, were also included in this study. Finally, the inner-membrane permeabilization was also monitored by uptake of the membrane-impermeable DNA-binding dye Sytox Green into *H. pylori* by measuring the increase in fluorescence due to drug administration. Exposure of cells to niclosamide (64 μg/mL) did not show any changes in cellular fluorescence, indicating that niclosamide does not cause any physical damage to *H. pylori* membranes (Fig. 7E).

### Cytotoxicity

**Figure 8:**
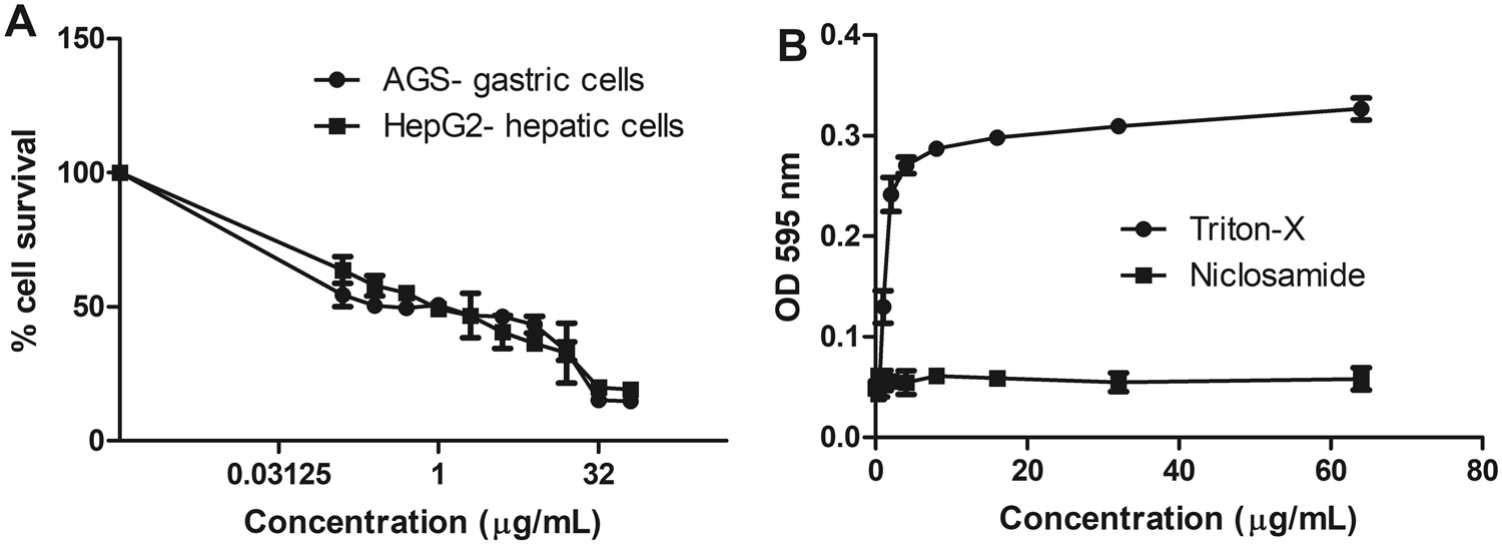
Cytotoxicity and hemolysis of niclosamide. (**A**) Cytotoxicity of niclosamide was assessed in HepG2 and AGS cells IC_50_ and was found to be 4 μg/mL. (**B**) Hemolysis of niclosamide was tested with h-RBC and we did not observe hemolysis. Data represent the mean ± SEM (n = 3).

Eventhough niclosamide is approved and widely used agent, we evaluated the cytotoxicity of niclosamide against a gastric cell line (AGS), as well as human red blood cells (h-RBC) and hepatic HepG2 cells. Serially diluted Triton-X (0.001 to 1%) was included as a positive control. As expected, niclosamide caused no hemolysis, while the IC_50_ against the gastric cell line, was similar to that of hepatic cells (Fig. 8A–C). Notably, niclosamide is not absorbed through the intestinal wall of the host and in murine models, animals survived even after a dose of 5 mg/kg^50^. The U. S. Environmental Protection Agency has reported that the human oral LD_50_ of niclosamide is 1 gm/kg and based on the IC_50_ values of niclosamide *in vitro*^51^, it seems to be cytotoxic. However, according to US EPA, the human oral LD_50_ is much higher^51^ and niclosamide could eradicate *H. pylori* from the gastric region with low cytotoxic level.

### The *in vivo* efficacy of niclosamide in the *Galleria mellonella* model

**Figure 9:**
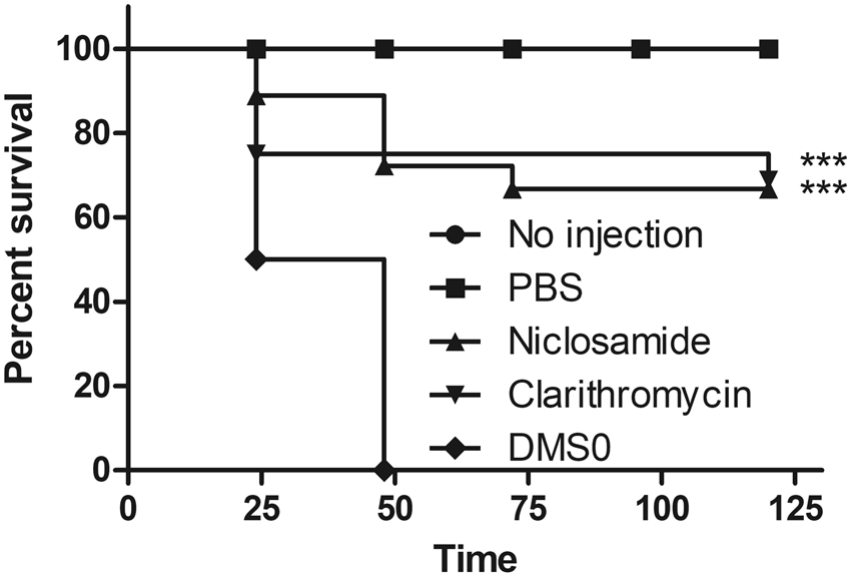
*In vivo* efficacy of niclosamide in the *G. mellonella* model. *H. pylori* were suspended in PBS (OD_600_ = 0.25) and 10 μL was injected into last left pro-leg of wax-moth. Niclosamide was administered at a concentration of 25 mg/kg and about 60% of wax-moth survived up to 120 h. Data depicts from two independent assays.

Giannouli *et al*., developed *G. mellonella* as an alternative model host for the evaluation of antimicrobial agents against *H. pylori* infection^52^. We monitored the surviving larvae every 24 hours, considering them dead if they lacked response to external stimuli. The niclosamide treatment significantly rescued larvae from *H. pylori* infection, with a survival rate up to 70% compared to the no treatment group (*p* < 0.0001) (Fig. 9). The clarithromycin injected group served as the positive control (up to 80% survival at 5 days).

## Conclusion

Repurposing is a potentially powerful approach to reveal unexplored antibacterial properties of existing clinical molecules. We found that niclosamide inhibits growth of *H. pylori* strain 60190 at low MIC and is effective against *H. pylori* at low pH. Combinatorial activity with clinical antibiotics showed an absence of antagonism. Niclosamide eliminated adhered/invaded *H. pylori* on gastric epithelial cells and decrease motility and IL-8 secretion. Interestingly, the MOA is mediated through the disruption of PMF and decrease of transmembrane pH. The *in vitro* and *in vivo* activity of niclosamide warrants future evaluation with other clinical strains and, eventually, evaluation in clinical trails.

## Methods

### Bacterial cultures

The *H. pylori* reference strain 60190 (ATCC 49503) was purchased from American Type Culture Collection (ATCC). Bacteria were cultured on Brucella agar (Becton Dickinson, Braintree, MA, USA) supplemented with 10% fetal bovine serum (FBS; Gibco, Long Island, NY, USA) and *H. pylori* selective supplement (vancomycin – 10.0 mg/l, cefsulodin – 5.0 mg/l, trimethoprim – 5.0 mg/l, amphotericin B – 5.0 mg/l) (Oxoid, Hampshire, UK) and maintained in humidified incubators at 37 °C under an atmosphere of 5% CO_2_.

### Antibacterial susceptibility assays

*In vitro* antibacterial activity was tested using the broth microdilution assay^53^. Experiments were carried out in triplicate using Müller-Hinton broth (BD Biosciences, Franklin Lakes, NJ, USA) supplemented with 10% FBS in 96-well plates (BD Biosciences) at a total assay volume of 100 μL. Antimicrobials used in the pilot study was selected from our previous HTS assay^14,21,54^ and tested against *H. pylori*. Anthelmentics (niclosamide, oxyclozanide, closantel, rafoxanide) were purchased from Sigma-Aldrich (St. Louis, MO, USA). Two-fold serial dilutions were prepared between the concentration range 0.01–64 μg/mL. An initial bacterial inoculum was adjusted to OD_600_ = 0.06 and incubated with test compounds at 37 °C for 3 days in humidified incubators under the 5% CO_2_ atmosphere. OD_600_ was measured and the lowest concentration of compound that inhibited bacterial growth was reported as the MIC^55^. The broth microdilution assay was used to demonstrate the stability of the niclosamide using MHB supplemented with 10% FBS. The pH was adjusted to acidic condition with 1 N HCl. The minimal bactericidal concentration (MBC) was determined by plating 5 μL of broth culture from the MIC assay onto Müller-Hinton agar (BD Biosciences) supplemented with 10% FBS. After 72 h, the lowest concentration at which colonies were not observed was reported as the MBC.

### Killing kinetic assays

The antibacterial properties of niclosamide against *H. pylori* were further examined using killing kinetic assays, as previously described^13^. Briefly, agar grown *H. pylori* bacteria were suspended in fresh MHB with 10% FBS to a density of 10^9^ cells/mL and placed into 10 mL tubes (BD Biosciences). Test compounds were then added at the 4× MIC and incubated with agitation at 37 °C under microaerophilic conditions. Aliquots were periodically drawn from the tubes, serially diluted and plated onto Brucella agar (BD Biosciences) supplemented with 10% FBS. CFUs were counted after a 3-day incubation and assays were carried out in duplicate.

### Checkerboard assays

Antibacterial synergy was tested using the checkerboard assay^56^. Niclosamide was combined with antibiotics and proton pump inhibitors (PPIs). In this series of experiments cultures of *H. pylori* were adjusted to OD_600_ = 0.06 and added to compound pairs that had been serially diluted in the same 96-well plates, vertically for one compound and horizontally for the other. Assays were carried out in triplicate as described in the antibacterial susceptibility assay sub-section. The combinatorial inhibitory concentration was indicated by the fractional inhibitory concentration index (FICI): FICI = MIC_**A**_ combination/MIC_**A**_ alone + MIC_**B**_ combination/MIC_**B**_ alone^56^.

### Adhesion and invasion assays

The AGS (Gastric adenocarcinoma cell lines) cell line was used to examine inhibition of adhesion and invasion of *H. pylori* by niclosamide, as described by Schmitt *et al*.^57^. The AGS cells were grown in DMEM (Gibco), supplemented with 10% fetal bovine serum (FBS) (Gibco) and 1% penicillin/streptomycin (Gibco) and maintained at 37 °C in 5% CO_2_^58,59^. Then, 5 × 10^5^ cells in antibiotic and serum-free DMEM were seeded in 6-well plates 24 h prior to infection. *H. pylori* bacteria at a multiplicity of infection (MOI) = 100 were added and allowed to adhere to the surface of the AGS cells. Planktonic bacteria were removed after 2 h and DMEM with niclosamide (1× MIC) was then added to the wells containing the AGS. The mammalian cells were lysed by 0.1% saponin after 20 h of incubation and the adhered bacteria were then serially diluted, plated in Brucella agar supplemented with 10% FBS, and incubated as described earlier. To determine the bacterial invasion inhibition, AGS cells were treated with DMEM supplemented with 200 μg/mL gentamicin and incubated for 1.30 h to eliminate extracellular bacteria. Antibiotic and serum-free DMEM with and without test compounds were added and incubated in 5% CO_2_ for 20 h. Cell lysate preparation, plating, and incubation were carried out as we described in the adhesion assay. Assays were carried out in triplicate^21,49^.

### Gene expression assays

To determine the effect of niclosamide in *H. pylori* VacA toxin expression, bacteria grown on agar were suspended in Brucella broth supplemented with 10% FBS (OD_600_ = 0.06) in dilutions of niclosamide (0, 100, 150, or 200 ng/mL) and incubated for 3 days^60^. After 3 days, the bacteria were washed and treated with TRIZOL (Invitrogen, Carlsbad, CA, USA), and the RNA concentration was determined using a NanoVue Plus spectrophotometer (GE Healthcare, Fairfield, CT, USA). PCR amplifications were performed and the products were analyzed by gel electrophoresis (1.0% agarose) containing SYBR^®^ Safe (Invitrogen). Gel images were captured and analyzed using the Quantity One System. To test whether niclosamide treatment inhibits *H. pylori-*induced IL-8 secretion, MKN-28 cells were co-cultured with *H. pylori* (MOI = 100) in the presence or absence of niclosamide. At 24 hours, RNA was isolated from the infected host cells. cDNA was synthesized using the Verso cDNA (Invitrogen) synthesis kit according to manufacturer instructions. Quantitative real-time reactions were carried out in a Bio-Rad iTaq universal SYBR (Bio-Rad, Hercules, CA USA) one-step kit and a CFX98 real-time PCR cycler^61^. The relative gene expression was calculated from Cq values using a ΔΔCq method. The primer sequences and PCR conditions are listed in Supplementary Table S2.

### Multi-step emergence of resistance

To determine whether *H. pylori* can develop resistance against niclosamide, bacterial cells were suspended in MHB with 10% FBS at OD_600_ = 0.06 in the presence of serially diluted niclosamide and incubated, as described in the antibiotic susceptibility assays sub-section. From the highest drug concentration allowing visible growth at sub-MIC concentration, aliquots were diluted (1:40) into fresh medium. These were used to inoculate the second set of serial drug dilutions as described by Dalhoff *et al*.^62^. After a 3 day incubation, *H. pylori* bacteria were passaged sequentially over 30 days and the OD was recorded.

### Motility assays

The motility assay was performed as described previously^63^. In brief, Brucella agar with 10% FBS was prepared in 2 layers. The bottom layer contained pre-casted 1.5% agar and the softer top layer was comprised of 0.4% agar and niclosamide (75, 100, 150 or 200 ng/mL). Agar-grown *H. pylori* cells were sliced and the densely grown *H. pylori* agar slice was placed facing up towards the soft layer and incubated as described in bacteria and mammalian cell culture subsection^64^.

### Scanning electron microscopy

*H. pylori* bacteria were suspended in Brucella broth with 10% FBS in the presence or absence or niclosamide (1x ,4x ,8x MIC) for 2 h under microaerophilic conditions with agitation. After 2 h, the bacterial cells were harvested and adhered to coverslips using a 1% poly-L-lysine (Sigma-Aldrich) solution. Then, cells were fixed in a 5% glutaraldehyde (Sigma-Aldrich), 4% paraformaldehyde (Sigma-Aldrich), 0.1 M sodium cacodylate (Sigma-Aldrich) solution. After fixation, the cells were washed in a 0.1 M sodium cacodylate buffer and dehydrated by immersion in increasing concentration of ethanol (from 30 to 100%). The critical point drying method (CPD) was applied to dehydrate the samples, the coverslips were mounted on aluminum stubs, and then sputter coated with gold (Emitech K550, Ashford, Kent, UK). Images were taken on a Hitachi 2700 Scanning electron microscope.

### Light microscopy imaging for vacuolation

AGS cells were harvested and seeded into a 6 well plate at a concentration of 5 × 10^5^ cells, 24 h before experimentation. *H. pylori* bacteria were harvested and washed with sterile PBS and co-cultured with AGS at a MOI of 100. After incubation for 24 h, the cells were washed, magnified under a microscope, and examined for vacuolation.

### Membrane permeabilization assays

Sytox Green (Life Technologies, Carlsbad, CA, USA) was used to probe the effects of niclosamide on *H. pylori* membrane permeabilization, as previously described^65^. Assays were carried out in duplicate in 96-well plates (Corning). Bacterial cells were re-suspended in phosphate buffered saline (PBS, pH 7.4) to OD_595_ nm = 0.2. Sytox Green was added at a final concentration of 5 μM and cells were incubated in the dark for 30 minutes. Cell suspensions (50 μL) were added to 50 μL of compound (64 μg/mL in PBS), and the fluorescence intensity was measured (excitation 485 nm, emission 530 nm) periodically over 60 minutes. DMSO was included as the vehicle control. Membrane disruption effects of compounds were indicated by an increase in cellular fluorescence caused by the enhanced permeability of the membrane impermeable DNA staining dye.

### Membrane potential

Membrane potential measurements were performed as described by Scott *et al*.^66^. In brief, *H. pylori* cells (OD_595_ nm = 0.2) were washed with a buffer (PBS, 100 mM KCl, 20 mM Glucose) and loaded with 5.0 μM of DISC3 (5) (Molecular Probes/Thermo Fisher Scientific, ON, Canada) dissolved in DMSO. The fluorescence was recorded (excitation 610 nm, emission 660 nm) periodically and valinomycin, amoxicillin, carbonyl cyanide *m*-chlorophenyl hydrazone (CCCP - a protonophore) were used as controls. The percentage of membrane potential change was calculated by comparing drug-treated cells with the untreated control, over all time points.

### Mammalian cell cytotoxicity assays

HepG2 and AGS cells were used to test the cytotoxicity of niclosamide, as described by Kwon *et al*.^67^. Cells were grown in DMEM (Gibco) supplemented with 10% fetal bovine serum (FBS) (Gibco) and 1% penicillin/streptomycin (Gibco) and maintained at 37 °C in 5% CO_2_. 5 × 10^4^ cells in 100 μL were added to wells of 96-well plates. Compounds were serially diluted in serum and antibiotic-free DMEM and added to the monolayer and incubated at 37 °C in 5% CO_2_ for 24 h. At 4 h prior to the end of the incubation period, 10 μL of 2-(4-iodophenyl)-3-(4-nitrophenyl)-5-(2, 4-disulfophenyl)-2*H*-tetrazolium (WST-1) solution (Roche, Mannheim, Germany) was added to each well. The WST-1 reduction was monitored at 450 nm using a Vmax microplate reader. Assays were performed in triplicate and the percentage survival was calculated by comparing the compound-treated wells to the DMSO-treated vehicle controls.

### Human blood cell (RBC) hemolysis assays

Human erythrocytes (Rockland Immunochemicals, Limerick, PA, USA) were used to measure the hemolytic activity of the compounds, as described by Isnansetyo *et al*.^68^ Human erythrocytes (4%, in PBS, 50 μL) were added to 50 μL of serially diluted test compounds in PBS in 96-well plates. After incubating at 37 °C for 1 h, the plates were centrifuged at 500 × g for 5 min and 50 μL of the supernatant from each well was transferred to a second 96-well plate. Absorbance (540 nm) was used as a measure of hemolytic activity. Assays were carried in triplicate.

### *G. mellonella* MRSA infection assay

Assays were performed as described by Giannouli *et al*.^52^. Twelve randomly selected *G. mellonella* larvae (Vanderhorst, Inc., St. Mary’s, OH, USA) between 300–350 mg were used for each group in the experiment. *H. pylori* cells were washed with PBS and diluted to OD_600_ nm = 0.3, before inoculation into *G. mellonella* larvae. A 10 μL inoculum was injected into the last left proleg using a 10 μL Hamilton syringe. After 2 h, compounds were administered at into the last right proleg and the wax moths were incubate at 37 °C. Three control groups - (1) injected with PBS only, (2) inoculated with *H. pylori* but treated with sham injections, (3) no manipulation - were included. *G. mellonella* survival was evaluated up to 120 h and considered dead if unresponsive to touch. Killing curves and differences in survival were analyzed by the Kaplan-Meier method using GraphPad Prism version 6.04 (GraphPad Software, La Jolla, CA, USA). Statistical analysis (Kruskal-Wallis test) was carried out using the same program.

### Statistical analysis

Statistical analysis (Two-way ANOVA followed by Bonfererroni post-test) was carried out using GraphPad Prism version 6.04 (GraphPad Software, La Jolla CA, USA) and *p* values of < 0.05 were considered significant.

### Data availability statement

No datasets were generated or analysed during the current study.

## Electronic supplementary material

Supplementary Information
