# Supplementary material for: Repurposing the anthelmintic drug niclosamide to combat Helicobacter pylori
Source: Sci Rep. 2018 Feb 27;8:3701. doi: 10.1038/s41598-018-22037-x (PMC5829259; doi:10.1038/s41598-018-22037-x)
Supplement: Supplementary file 1 — Supplementary Information [file 41598_2018_22037_MOESM1_ESM.pdf]

Repurposing the anthelmintic drug niclosamide to combat *Helicobacter pylori*

Nagendran Tharmalingam[1], Jenna Port[1], Dawilmer Castillo[1], Eleftherios Mylonakis[1, *]

[1]Infectious Diseases Division, Warren Alpert Medical School of Brown University, Rhode Island Hospital, Providence, RI, 02903, USA.

***Corresponding author:** Eleftherios Mylonakis, M.D., Ph.D., FIDSA,

Rhode Island Hospital

593 Eddy Street, POB, 3rd Floor, Suite 328/330

Providence, RI, 02903

Tel: 401-444-7856

Fax: 401-444-8179

Email: emylonakis@lifespan.org

Supplementary Table S1. List of Primers

Primer	Relevant sequence	Size	Amplification cycles	Annealing temperature	References
***vac*A-F**	5' AAACGACAAGAAGAGATCAGT 3’	291	20	58	[51,63]
***vac*a-R**	3’ CCAGCAAAAGGCCCATCAA 5'				
***gal*E-F**	5' ATGGCATTATTATTCACAGG 3’	461	20	58	[51]
***gal*E-R**	3’ GCTCCATAAGGATTAATGGG 5'				
	Real-Time PCR Primers				
**IL 8- F**	5'-ACT GAG AGT GAT TGA GAG TGG AC-3		40		[64]
**IL 8–R**	5'-AAC CCT CTG CAC CCA GTT TTC-3'				
**18 rRNA–F**	5'-CGG CGA CGA CCC ATT CGA AC-3'		40		[64]
**18s rRNA-R**	5'-GAA TCG AAC CCT GAT TCC CCG TC-3'				

Supplementary Table S2. Pilot screening of hit compounds from MRSA and *F. tularensis* LVS screen for the anti-*H. pylori* activity.

Antibacterials (Hit molecules from MRSA and *F. tularensis*LVS screen)	MIC	Structure
1	1	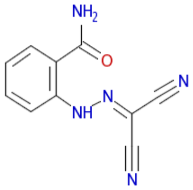
2	1	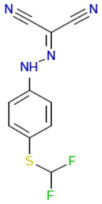
3	1	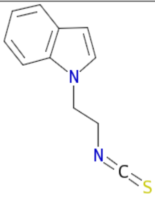
4	2	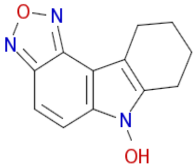
5	4	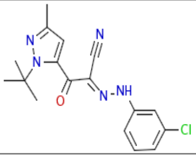
6	4	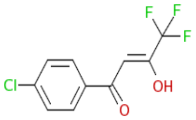
7	8	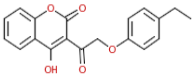
8	16	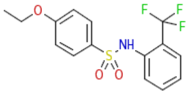
9	16	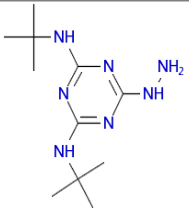

10	32	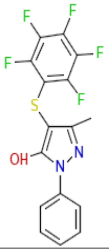
11	32	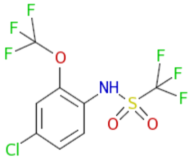
12	64	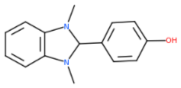
Apramycin	2	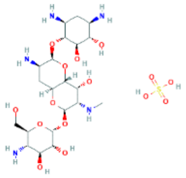
Mefloquine	8	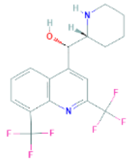
Paroxetine	16	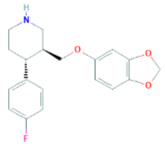
Diflunisal	32	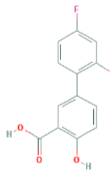

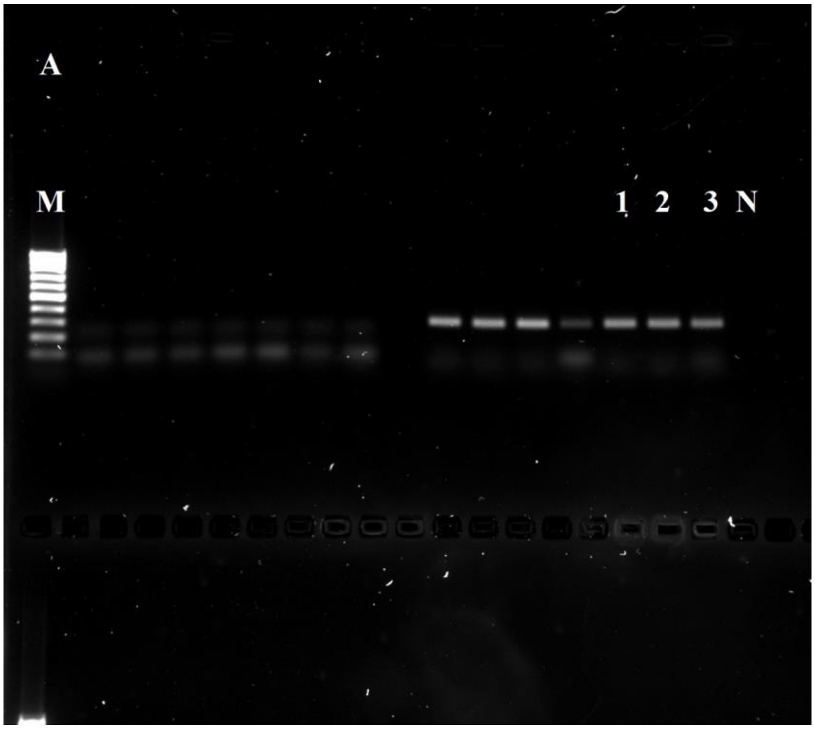

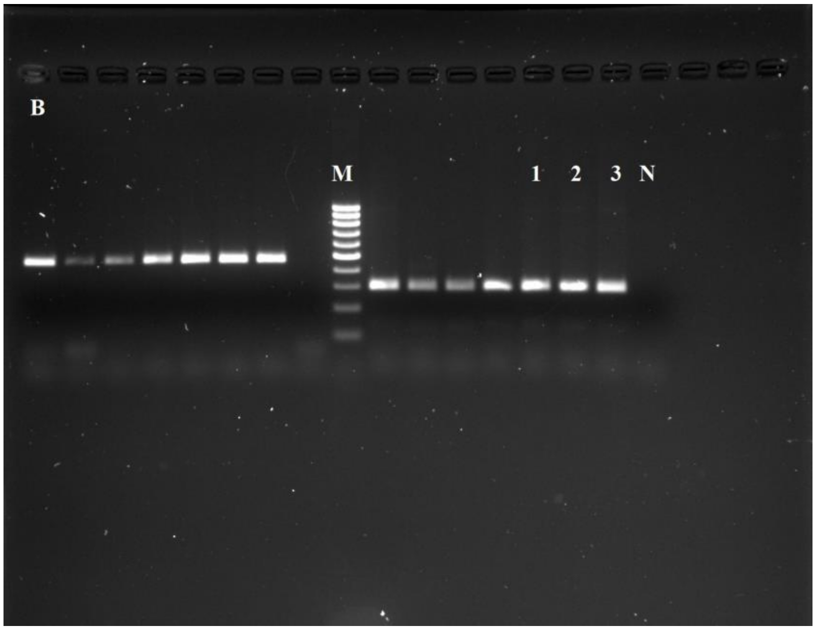

Supplementary Figure S3. Uncropped full-length gel picture. S3 A. Niclosamide down-regulated *vacA* gene (291 bp) expression at sub-MIC (0.2 µg/mL). M – Marker, 1. DMSO; 2. Niclosamide- 150 µg/mL; 3. Niclosamide- 200 µg/mL; N. Negative control. **S3 B**. Normalization of house-keeping gene *rpoB* (301 bp) in niclosamide treatment. M – Marker, 1. DMSO; 2. Niclosamide- 150 µg/mL; 3. Niclosamide- 200 µg/mL; N. Negative control.

51 Tharmalingam, N. *et al.* Piperine treatment suppresses Helicobacter pylori toxin entry in to gastric epithelium and minimizes β-catenin mediated oncogenesis and IL-8 secretion in vitro. *American journal of translational research* **8**, 885 (2016).

63 Boonjakuakul, J. K., Canfield, D. R. & Solnick, J. V. Comparison of Helicobacter pylori virulence gene expression in vitro and in the Rhesus macaque. *Infection and immunity* **73**, 4895-4904 (2005).

64 Tsai, K.-W. *et al.* Difference in the regulation of IL-8 expression induced by uropathogenic E. coli between two kinds of urinary tract epithelial cells. *Journal of biomedical science* **16**, 91 (2009).
